# Incidence and Time-to-Onset of Carbapenemase-Producing Enterobacterales (CPE) Infections in CPE Carriers: a Retrospective Cohort Study

**DOI:** 10.1128/spectrum.01868-22

**Published:** 2022-11-02

**Authors:** B. Hoellinger, S. Deboscker, F. Danion, T. Lavigne, F. Severac, Y. Ruch, A. Ursenbach, N. Lefebvre, P. Boyer, Y. Hansmann

**Affiliations:** a Department of Infectious and Tropical Diseases, Hôpitaux Universitaires de Strasbourg, Strasbourg, France; b Service d’Hygiène Hospitalière, Hôpitaux Universitaires de Strasbourg, Strasbourg, France; c Department of Bacteriology, Hôpitaux Universitaires de Strasbourg, Strasbourg, France; d Service de Réanimation Médicale de Hautepierre, Hôpitaux Universitaires de Strasbourg, Strasbourg, France; e Groupe Méthodes en Recherche Clinique (GMRC), Hôpital Civil, Hôpitaux Universitaires de Strasbourg, Strasbourg, France; Emory University School of Medicine

**Keywords:** carbapenemase-producing Enterobacterales (CPE), mortality, rectal carriage, risk factors, bacterial resistance

## Abstract

This study aimed to assess the proportion of carbapenemase-producing Enterobacterales (CPE) infections among all infectious episodes in CPE carriers, compare the time-to-onset of CPE infections with that of other infections, assess the mortality of patients with CPE infections, and identify risk factors for CPE infections in CPE carriers. A retrospective cohort study was performed over a 10-year period in our University Hospital, and 274 CPE carriers were identified. All infectious episodes within the first 6 months following the diagnosis of CPE rectal carriage were considered. Risk factor analysis for CPE infections in CPE carriers was performed by univariate and multivariate analyses. This study revealed an incidence of 24.1% (66/274) of CPE infection within 6 months of CPE carriage diagnosis. The 28-day all-cause mortality due to CPE infections was 25.7%. CPE infections represented 52.6% (70/133) of all infectious episodes in CPE carriers in the first 6 months following CPE carriage detection, and these significantly occurred earlier than non-CPE infections, with a median time of 15 versus 51 days, respectively (*P* < 0.01). Based on the multivariate analysis, prior neurological disease was the only risk factor associated with CPE infections in CPE carriers. CPE infections have an early onset, accounting for a large proportion of infections in CPE carriers, and are associated with high mortality.

**IMPORTANCE** Carbapenemase-producing Enterobacterales (CPE) infections are emerging infections and may represent a therapeutic challenge, while effective antibiotic therapy is likely to be delayed. We aimed to assess the proportion of CPE infections in CPE carriers and to identify risk factors of CPE infections among this population that could guide empirical antibiotic therapy. We showed that CPE infections are frequent in CPE carriers, have an early onset after CPE carriage diagnosis, and represent a significant proportion of all infectious episodes in CPE carriers. No significant risk factors for CPE infections could be identified. Overall, this study suggests that empirical antibiotic treatment covering CPE might be initiated in CPE carriers at least in the first month after its diagnosis and in severe infections due to the high frequency and early occurrence of CPE infections in these patients.

## INTRODUCTION

Carbapenemase-producing Enterobacterales (CPE) infections are emerging infections in Europe and may represent a therapeutic challenge. CPE carriage is associated with travel and hospitalization in areas with endemic or epidemic circulation of these strains, inappropriate or recurrent antibiotic therapies, and insufficient compliance to standard hygiene precautions (1, 2).

Unlike infections in extended-spectrum beta-lactamase-producing (ESBL) Enterobacterales carriers, there is no guideline for the empirical antibiotic treatment of infections in CPE carriers (3). This study aimed to assess the proportion of CPE infections among all infectious episodes in CPE carriers, compare the time-to-onset of CPE infections with that of other infections, assess the mortality of patients with CPE infections, and identify risk factors for CPE infection in CPE carriers.

## RESULTS

### CPE rectal carriage: patient characteristics.

**FIG 1:**
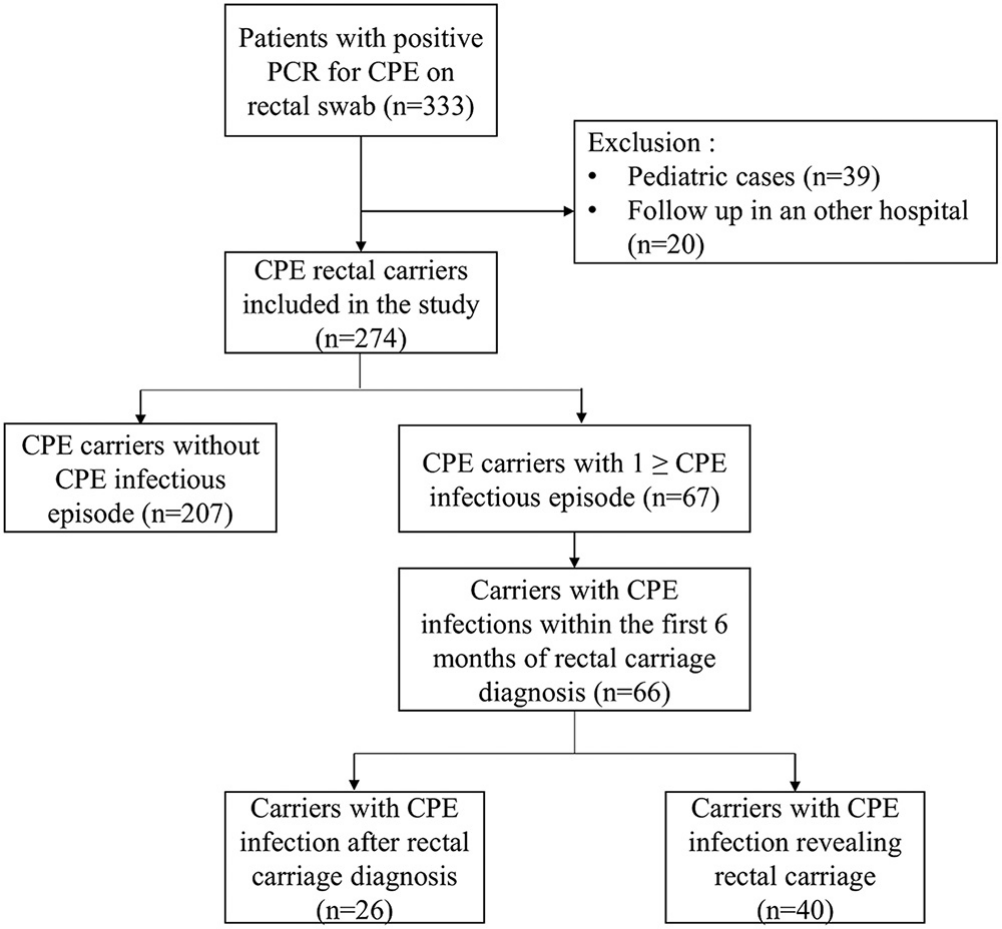
Flowchart.

Between January 1, 2011 and December 30, 2021, 333 CPE carriers were identified; 39 pediatric cases and 20 patients who were affiliated in another hospital in the area were excluded. A total of 274 patients were included in the study (Fig. 1). Among them, 226 (82.5%) were identified between 2017 and 2021 following epidemics in several hospital intensive care units (ICU) and surgery departments.

The patients’ ages ranged from 19 to 99 years (median age, 66), and 102 (37.2%) were female. CPE rectal carriage was diagnosed in 47.8% of patients (131/274) in medical departments, 28.8% of patients (79/274) in ICUs, and 23.3% of patients (64/274) in surgery departments (Table 1).

**TABLE 1:** Characteristics of the CPE rectal carriers

Variables	CPE rectal carriers
Age (yrs), median (extremes)	66 (19 to 99)
Sex, female	102 (37.2)
BMI ≥30kg/m²	62 (22.6)
Smoking	60 (21.9)
Chronic alcoholism	33 (12.0)
Diabetes mellitus	96 (35.0)
HTA	160 (58.4)
Prior cardiac disease	72 (26.3)
Prior respiratory disease	45 (16.4)
Prior neurological disease	54 (19.7)
Cirrhosis	20 (7.3)
Chronic kidney failure Including moderate to severe	88 (32.1) 75 (85.2)
Hemodialysis	15 (5.5)
Hematologic malignancies	27 (9.9)
Allogenic or autologous blood stem cell transplantation	9 (3.3)
Solid tumor	46 (16.8)
Including digestive tumor	28 (61.0)
Urological history excluding urinary catheter	17 (6.2)
Solid organ transplantation Liver Kidney	31 (11.3) 17 (54.8) 12 (38.7)
Chronic inflammatory disease	18 (6.6)
Systemic steroids use	19 (6.9)
Other immune defects	5 (1.8)
Charlson Comorbidity Index. Mean ± SD	4.5 ± 2.8
≥4 comorbidities	96 (35.0)
Hospital stay in the previous 6 mo	252/268 (94.0)
ICU stay in the previous 6 mo	126/262 (48.1)
Diagnosis of carriage	
ICU Medical department Surgery department	79 (28.8) 131 (47.8) 64 (23.3)
Surgery in the past 3 mo Including abdominal surgery	78/266 (29.3) 46 (59.0)
Travel abroad in the past yr	49 (17.9)
Antibiotics in the previous 6 mo Including carbapenems in the previous 6 mo	191/254 36 (18.8)

### CPE rectal carriage: microbiological characteristics.

A VIM carbapenemase was identified in 128/274 (46.7%) carriers, an OXA-48-like carbapenemase in 114/274 (41.6%) carriers, a NDM carbapenemase in 33/274 (12.0%) patients, and a KPC carbapenemase in 7/274 (2.7%) patients. These numbers included eight patients in whom multiple carbapenemases were identified: NDM and OXA-48-like carbapenemases in four carriers, VIM and OXA-48 carbapenemases in two carriers, and NDM and VIM and OXA-48-like carbapenemases in one carrier.

Klebsiella pneumoniae and Enterobacter cloacae complexes were isolated in 93/274 (33.9%) and 92/274 (33.6%) patients, respectively, while Escherichia coli and Citrobacter koseri complexes were isolated in 75/274 (27.4%) and 46/274 (16.8%) patients, respectively. The other isolated species included Citrobacter amalonaticus, Klebsiella aerogenes, Klebsiella oxytoca, Klebsiella variicola, Proteus mirabilis, Raoultella planticola, Morganella morganii, and *Serratia marsescens.* Among these patients, 62/274 (22.6%) had rectal swabs with >1 species of CPE.

### CPE infections: microbiological characteristics.

Considering the first CPE infectious episode in each patient (e.g., not considering the recurrences), Klebsiella pneumoniae was isolated in 33 (42.9%) CPE-infectious episodes, followed by Enterobacter cloacae complex in 22 episodes (28.6%) and E. coli in 10 episodes (13.0%). VIM carbapenemase was isolated in 39 (48.8%) clinical samples, while OXA-48-like, NDM, and KPC carbapenemases were detected in 30 (37.5%), nine (11.1%), and two (3.7%) clinical samples, respectively. MIC50 for meropenem and imipenem were 0.5 and 2.0 mg/L, respectively, for VIM producing isolates. MIC90 for meropenem and imipenem were 1.5 and 24.0 mg/L, respectively, for VIM-producing strains, and 24.0 and 16.0 for OXA-48 producing strains.

### CPE infections: clinical characteristics.

Among the 274 CPE carriers, 66 (24.1%) patients developed at least one CPE infection in the first 6 months following the diagnosis of CPE carriage. In total, 70 infectious episodes were identified in the first 6 months.

Four (6.1%) patients had a CPE infection recurrence. In 40/66 (60.6%) patients, CPE infections revealed the CPE rectal carriage.

Among 70 CPE infections, bacteremia and pneumonia represented the most common infections with 33 (47.1%) and 17 (24.3%) infectious episodes, respectively, while biliary/digestive tract infections, urinary tract infections. and osteoarticular infections without bacteremia had represented six infectious episodes (8.6%) each.

### Proportion of CPE infections among all infections in CPE carriers.

**FIG 2:**
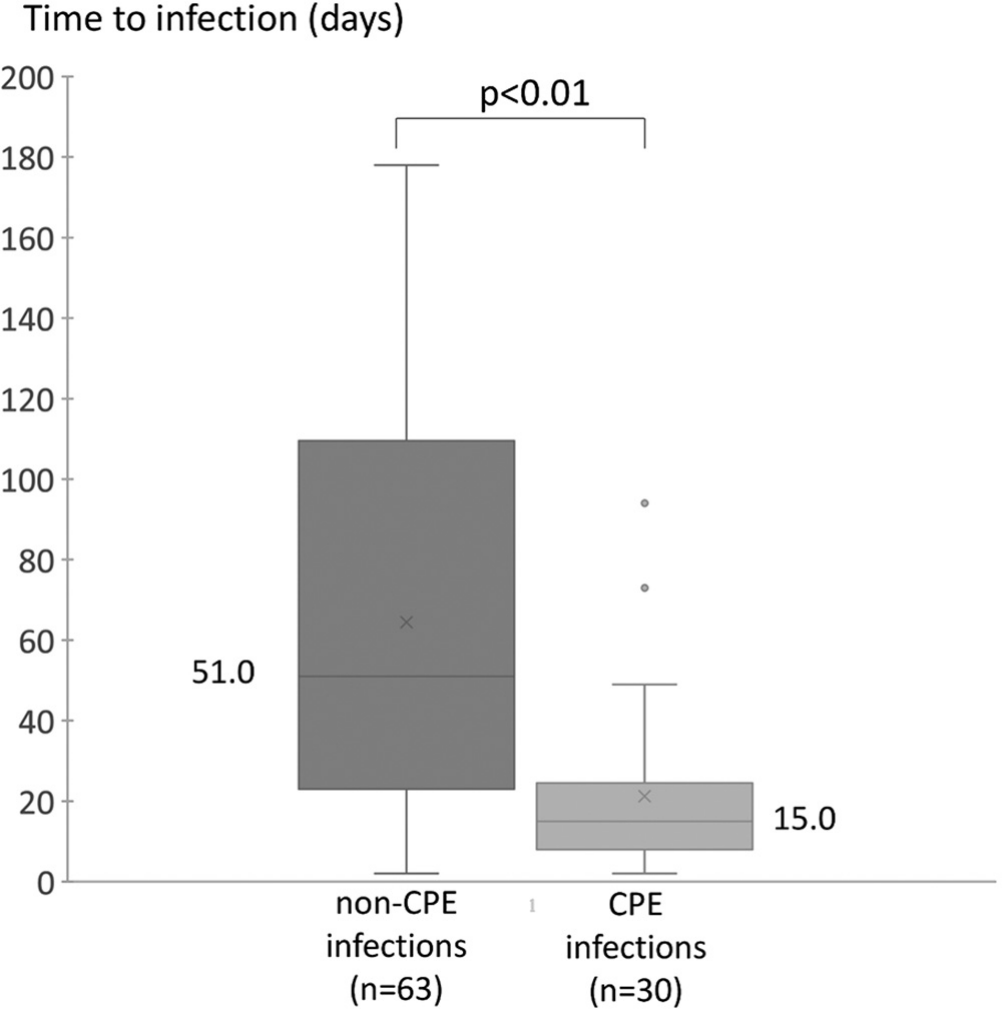
Time to onset of infections in CPE carriers. “Non-CPE infections” refers to all infections others than those due to CPE.

Excluding the 40 infectious episodes that revealed the CPE carriage, 93 infectious episodes were identified in the first 6 months following its diagnosis (Fig. 2). Infections involved CPE in 30/93 cases (32.3%) while Enterobacterales not producing carbapenemases caused 23/93 infections (24.7%), including eight ESBL Enterobacterales and three high-level cephalosporinase-producing Enterobacterales. Other bacteria caused 22/93 infections (23.7%). Infections were not microbiologically documented in 18/93 (19.4%) cases: five aspiration pneumonias, five neutropenic fevers, three chronic obstructive pulmonary disease exacerbations with a presumed infectious trigger, two infectious syndromes in solid organ transplant recipients, one cholangitis, one cellulitis, and one cyst infection in a patient with polycystic kidney disease.

When including the 40 infections that revealed the CPE carriage, CPE infections represented 52.6% (70/133) of all infectious episodes in the first 6 months.

### Time-to-onset of infection in CPE carriers.

Among the 234 CPE carriers (excluding the infectious episodes that revealed the CPE carriage), CPE infection occurred earlier than any other infections (*P* < 0.01), with a median of 15 days (Q1 to Q3 = 8 to 24) compared with 51 days (Q1 to Q3 = 25 to 109) (Fig. 2). No other infections occurred between CPE carriage detection and CPE infection.

### Mortality.

The 28-day all-cause mortality in the case of CPE infection was 25.7% (17/66). The mortality rate due to CPE bacteremia was 28.1% (9/33), while that due to CPE infections in ICUs was 35.7% (10/28).

### Risk factors for CPE infection in CPE carriers.

When considering all CPE carriers, 96/274 (35.0%) had ≥4 comorbidities, and 252/268 (94.0%) were hospitalized in the previous 6 months.

There was no difference in antibiotic exposure in the 6 months preceding the diagnosis of CPE carriage in patients who developed an infection compared with carriers who did not; 75.1% (191/235) of the patients received antibiotics in the previous 6 months, including 18.8% (36/191) who received carbapenems. No significant difference in age, comorbidities, and length of ICU stay in the previous 6 months was observed when comparing carriers who developed a CPE infection to those who did not (Table 2).

**TABLE 2:** Comparison of the characteristics between CPE carriers who developed and did not develop CPE infection^*a*^^,^^*b*^

Variables	CPE carriers with CPE infection (*n* = 66)	CPE carriers without CPE infection (*n* = 208)	*P* value
Age (yrs), median (IQR)	61.0 (48.8 to 75.0)	67.0 (56.0 to 76.0)	0.11
Sex, female	45 (68.2)	126 (60.6)	0.27
BMI mean (IQR) (kg/m²)	26.0 (21.7 to 30.5)	25.8 (22.3)	0.86
Smoking	13 (19.7)	47 (22.6)	0.62
Diabetes mellitus	23 (34.8)	73 (35.1)	0.97
HTA	37 (56.1)	123 (59.1)	0.66
Prior cardiac disease	17 (25.8)	55 (26.4)	0.91
Prior respiratory disease	8 (12.1)	37 (17.8)	0.23
Prior neurological disease	7 (10.6)	47 (22.6)	0.03
Cirrhosis	6 (9.1)	14 (6.8)	0.53
Chronic kidney failure Including moderate to severe	20 (30.3) 15 (75.0)	68 (32.7) 60 (88.2)	0.72
Hemodialysis	3 (4.5)	12 (5.8)	1.00
Hematologic malignancies	9 (13.6)	18 (8.7)	0.24
Allogenic or autologous blood stem cell transplantation	4 (6.1)	5 (2.4)	0.15
Solid tumor Including digestive tumor	13 (19.7) 9 (13.6)	33 (15.9) 19 (9.1)	0.47
Urological history excluding urinary catheter	4 (6.1)	13 (6.2)	0.96
Solid organ transplantation Liver Kidney	8 (12.1) 7 (87.5) 1 (12.5)	23 (11.1) 10 (43.5) 11 (47.8)	0.81
Chronic inflammatory disease	4 (6.1)	14 (6.7)	0.85
Systemic steroids use	9 (13.6)	10 (4.8)	0.01
Other immune defects	2 (3.0)	3 (1.4)	0.40
Charlson comorbidity index. mean ± SD	4.00 (2.25, 6.00)	4.00 (2.50, 6.00)	0.51
≥4 comorbidities	26 (39.3)	70 (33.7)	0.39
Hospital stay in the previous 6 mo	60 (92.3)	192 (94.6)	0.50
Duration of hospitalization, days, mean (1st and 3rd quartiles)	23.00 (11.00, 44.50)	24.50 (13.25, 41.75)	0.89
ICU stay in the previous 6 mo Length of stay in ICU, days, mean (IQR)	30 (47.6) 23.00 (11.00, 44.50)	96 (48.2) 24.50 (13.25, 41.75)	0.93 0.89
Diagnosis of carriage in ICU	26 (39.4)	53 (25.5)	0.03
ICU stay	11 (22.9)	28 (42.4)	0.03
Surgery in the past 3 mo Including abdominal surgery	22 (33.3) 8 (36.3)	56 (28.0) 38 (67.9)	0.41 0.72
Bacteraemia in the past mo Including Gram-negative bacteria	15 (22.7) 8 (53.3)	20 (9.8) 11 (55.0)	0.01
Travel abroad in the past yr	14 (21.2)	37 (18.2)	0.59
Antibiotics in the previous 6 mo Including carbapenems in the previous 6 mo	50 (79.4) 10 (20.0)	140 (73.7) 26 (18.6)	0.37 0.82
Mean no. of days of antibiotics in the previous 6 mo ± SD	
Amoxicillin-clavulanic acid Piperacillin-tazobactam Carbapenems Ceftriaxone/cefotaxime Ceftazidime Cefepime Aminoglycoside Fluoroquinolone Glycopeptides/Lipopeptides Linezolid Systemic antifungal	1.7 ± 3.8 5.7 ± 8.3 2.1 ± 5.9 4.1 ± 7.2 0.7 ± 3.9 0.2 ± 1.3 0.6 ± 2.1 2.6 ± 5.8 3.1 ± 7.5 2.6 ± 5.2 2.3 ± 6.9	1.5 ± 3.2 4.6 ± 6.9 1.8 ± 6.3 3.3 ± 6.2 0.7 ± 3.0 0.4 ± 2.7 0.7 ± 2.0 2.3 ± 5.2 3.2 ± 7.6 2.0 ± 5.1 2.2 ± 6.3	0.86 0.47 0.67 0.78 0.81 0.57 0.24 0.44 0.91 0.11 0.85
3GCR *Enterobacteriaceae* carriage	10 (15.2)	40 (19.5)	0.43
CPE carriage diagnosis before changes in screening methods	17 (25.8)	34 (16.3)	0.087

a 3GCR, third-generation cephalosporin resistant; BMI, body mass index; IQR, interquartile range.

b Data are in *n* (%), unless stated otherwise.

In the multivariate analysis presented in Table 3, only prior neurological disease was significantly associated with a significant risk for CPE infections in carriers (*P* = 0,015).

**TABLE 3:** Multivariate analysis of risk factors for infection in CPE carriers

Variables	OR (CI 95%)	*P*
Systemic steroids use	1.218 (0.260 to 5.709)	0.802
Diagnosis of CPE carriage in ICU	0.622 (0.143 to 2.700)	0.526
Bacteraemia in the past mo	2.524 (0.696 to 9.155)	0.159
ICU stay	4.148 (0.917 to 18.752)	0.065
CPE carriage diagnosis before changes in screening methods	0.257 (0.086 to 0.770)	0.302
Prior neurological disease	1.689 (0.625 to 4.565)	0.015

## DISCUSSION

The empirical treatment of infections in CPE carriers was not addressed in the recent IDSA and ESCMID guidelines on the treatment of CPE infections (3). If the antimicrobial spectrum should include CPE, such treatment would require broad-spectrum antibiotics with a significant ecological impact and possible toxicity. By focusing on the proportion of CPE infections in carriers and the time to onset of CPE infection, our study may provide some answers.

In our cohort, 24.1% of the patients developed at least one CPE infection in the first 6 months, and the median time from CPE carriage diagnosis to CPE infection was 15 days. Only patients in whom the CPE carriage was known before CPE infection diagnosis were taken into account to calculate time to infection. However, these results are consistent with those of previous reports. A systematic review of literature in 2016 estimated that 16.5% of CPE carriers develop an infection, whereas a recent case-control study identified 37.1% of infections in CPE carriers (4, 5). In previous studies, the median time between the diagnosis of CPE carriage and CPE infections was between 11 and 20 days (6, 7).

The 28-day all-cause mortality of 25.7% in cases with CPE infections was lower than that reported in other studies. A 2018 meta-analysis reported an all-cause mortality within 30 days of 41.0% in cases of KPC infection, while a prospective study reported a 50% mortality in cases of bacteremia due to OXA-48 CPE (8, 9). There might be two explanations to this discrepancy. First, the VIM CPE isolates in our hospital are usually still susceptible to carbapenems, especially imipenem and meropenem, which are often used for the empirical treatment of health care-associated infections. Moreover, these studies were carried out prior to the marketing of new β-lactam-β-lactamase inhibitor combinations, such as ceftazidime-avibactam or meropenem-vaborbactam, which could reduce the mortality due to OXA-48 or KPC CPE infections compared with other treatments (10–12).

We could not identify significant risk factors for CPE infections in carriers. The high number of comorbidities and frequent hospitalizations with exposure to multiple antibiotics for most CPE carriers limit the identification of risk factors of infections for daily practice. Length of ICU stay and venous catheters are often identified as risk factors (5–7). The presence of invasive devices like urinary catheter or central venous access could not be collected due to the follow-up period of 6 months, multiple hospitalizations, and due to the retrospective design of this study. On the contrary, some risk factors for CPE infections in carriers are inconstantly identified from one study to another: coma, diabetes, radiotherapy and chemotherapy, invasive procedures, including abdominal surgery, prior antibiotic exposure, and invasive devices, such as tracheotomies and urinary catheters (5–7, 13).

We found no other study that assessed the proportion of CPE infections among all infectious episodes in CPE carriers and compared the time-to-onset of CPE infections with that of other infections. This study included patients hospitalized in medical wards, surgical wards, and ICUs, whereas most published studies regarding CPE carriers focused on those only in ICUs.

Aside from the retrospective and single-center nature of this study, another limit is the possible underestimation of the number of CPE carriers or CPE infections because a low carbapenem hydrolysis by VIM carbapenemase might hamper their detection (14). Thus, the nonsystematic screening for CPE carriage was not performed in our hospital; it focused on patients who stayed abroad, contact patients, and ICU patients. Another limit might be the change in screening methods over time, but CPE outbreak in our institution occurred mainly after 2017 with only sporadic cases in the previous years. In addition, screening methods have not undergone significant changes since 2017. Additionally, tertiary care centers receive patients that require a higher level of care, especially intensive care, which may limit the generalizability of our results to secondary care centers, especially the estimation of the risk of infection in CPE carriers. Finally, due to the retrospective nature of the study, infections and colonization without infection might have been misclassified even if consensual diagnosis criteria were applied.

In conclusion, CPE infections have an early onset, accounting for a large proportion of infections in CPE carriers, and are associated with high mortality. Overall, this study suggests that empirical antibiotic treatment covering CPE might be initiated in CPE carriers at least in the first month after its diagnosis and in severe infections due to the high frequency and early occurrence of CPE infections in these patients. The choice of empirical therapy should also be tailored to a local ecology. All patients included in our study exhibited CPE rectal carriage: these issues need to be considered when interpreting study data. Additional prospective studies are needed to determine which patients might benefit the most from such empirical therapy. Such studies could include a control arm of non-CPE carriers. Fecal microbiota transplantation is an emerging strategy to reduce antibiotic-resistant bacteria colonization and might be a future option to prevent CPE infections in CPE carriers (15).

## MATERIALS AND METHODS

### Setting and design.

The University Hospital of Strasbourg is a 2,158-bed tertiary care teaching hospital in France. This hospital has an ongoing CPE outbreak since 2011 (mainly VIM- and OXA-48-like producing Enterobacterales). A retrospective cohort study was performed from January 2011 to December 2020. The study population included patients who were identified as CPE carriers. In our hospital, rectal carriage screening is performed on patients who travelled or were hospitalized abroad, on patients in contact with CPE carriers, and in most hospital intensive care units (ICU). The exclusion criteria were patients <18 years and those affiliated with other hospitals in the area.

### Data collection.

Data were collected from computerized medical records. Rectal carriers were identified by extracting positive PCRs on rectal swabs. CPE infections in CPE carriers during the entire study period were identified, including in patients who were retrospectively found to have CPE carriage. Patient outcomes (28-day all-cause mortality), demographic characteristics, comorbidities, and antibiotic exposure (type of antibiotic and treatment duration) within 6 months preceding CPE carriage and all infectious episodes within 6 months after its diagnosis were collected, including community-acquired infections and usually undocumented infections (community-acquired pneumonia, aspiration pneumonia, cholecystitis, colitis, cellulitis, febrile neutropenia, culture negative sepsis without clear source, cyst infection in polycystic kidney disease). Bacteraemia in the month preceding CPE carriage diagnosis, regardless of the bacterium, were collected. The local ethics committee approved this study (reference CE-2021-78).

### Statistical analysis.

**(i) Risk factors for CPE infections.** Categorical variables are presented as sample size and percentages, while continuous data are presented as median, first, and third quartiles. Continuous variables were compared using Mann–Whitney U test. Categorical variables were compared using Pearson’s χ^2^ or Fisher’s test. The variables associated with CPE infection were identified using a multivariable logistic regression model which include the variables presenting a *P* value less than 0.1 in univariate analysis. The results are presented as odds ratios and 95% confidence intervals. A *P* value less than 0.05 is considered statistically significant. Statistical analyses were performed using the R Software version 4.1.1.

**(ii) Time-to-onset of CPE infections.** Patients were considered from the day of CPE carriage diagnosis up to 6 months, or before in case of death. Time-to-onset of CPE infections was compared with that of other infections using the Wilcoxon signed-rank test.

### Microbiological methods.

Rectal swabs were streaked onto two selective agar plates (chromID CARBA SMART and chromID BLSE [bioMérieux, Marcy l’étoile, France]) for a specific request of CPE carriage screening. In the absence of this specific request, only the chromID BLSE agar plate was inoculated.

For each colony morphotype, identification was carried out using a Microflex MALDI-TOF mass spectrometer (Bruker, Bremen, Germany). For each isolated colony of Enterobacterales, disk diffusion tests were performed on Mueller–Hinton (MH) and MH plus oxacillin (250 mg/L) agar plates. In case of decreased sensitivity to ertapenem or imipenem, an algorithm from the French National Reference Center for antibiotic resistance was applied to rule out most cases of resistance due to decreased permeability (16). The Xpert Carba-R panel on the GeneXpert system from Cepheid was used to identify the carbapenemase.

From 2011 to 2014, CPE selective agar plates as chromID CARBA SMART were not available in our laboratory and only chromID BLSE agar plates were inoculated. From 2011 to 2016, molecular identification of the CPE was carried out at the French National Reference Center for Antibiotic Resistance (Bicêtre Hospital, Paris). Between 2017 and 2021, CPE screening methods have not undergone significant changes.

For clinical samples, bacteria were identified by mass spectrometry. Antimicrobial susceptibility testing (AST) was performed on colonies from urine samples using Vitek 2 (bioMérieux, Marcy l’étoile, France). In case of a decreased susceptibility to ertapenem, MICs of ertapenem, meropenem and imipenem, and disk diffusion AST were performed on MH and MH plus oxacillin (250 mg/L) agar plates. For other samples, AST was performed by disk diffusion on an MH agar, as mentioned above. AST interpretation was performed according to EUCAST breakpoints.

### Definitions.

A CPE-positive peripheral blood culture defined a bacteremia. The diagnosis of catheter-related bacteremia was made if the differential of time-to-positivity between blood cultures taken from the central line and peripheral blood culture was longer than 2 h (17). In case of a negative peripheral blood culture and CPE-positive central blood culture, a diagnosis of infection or colonization of the central venous access was made.

Ventilation-acquired pneumonia (VAP) caused by CPE was diagnosed if the Centers for Disease Control and Prevention (CDC) criteria for VAP were met and if a CPE was isolated from respiratory samples with a quantitative culture above the diagnostic threshold of 10^4^ UFC/mL for bronchoalveolar lavage (BAL) and 10^5^ UFC/mL for endotracheal aspiration (18).

Urinary tract infection was diagnosed based on clinical symptoms associated with a significant colony concentration in urinary samples, as defined in the European guidelines for urinalysis (19).

The day of the collection of the first CPE-positive rectal samples defined the first day of colonization. The day of the collection of the first positive microbiological sample defined the first day of infection. In case of undocumented infections, such as aspiration pneumonia, the date of the first clinical signs defined the first day of infection.

Infection recurrence was defined as a culture with the same microorganism and reoccurrence of symptoms after completion of the antibiotic treatment and clinical resolution of the index infection.
